# Blush in Lung Contusions Is Not Rare and Has a High Risk of Mortality in Patients With Blunt Chest Trauma

**DOI:** 10.3389/fmed.2022.858511

**Published:** 2022-06-09

**Authors:** Naoki Tominaga, Mineji Hayakawa, Shoji Yokobori

**Affiliations:** ^1^Department of Emergency and Critical Care Medicine, Nippon Medical School Hospital, Tokyo, Japan; ^2^Department of Emergency Medicine, Hokkaido University Hospital, Sapporo, Japan

**Keywords:** lung contusion, pulmonary contusion, blush, chest trauma, contrast enhanced computed tomography, blunt trauma

## Abstract

**Background:**

Patients with blunt chest trauma have a high mortality rate. The assessment of blush in hepatic and splenic trauma is important for determining the need for emergency hemostatic interventions. However, the frequency and importance of blush in lung contusions are unknown. Therefore, this study aimed to evaluate the frequency of blush in patients with lung contusions and elucidate the relationship between blush and the clinical outcomes of patients with blunt chest trauma.

**Materials and Methods:**

In this retrospective observational study, we enrolled patients with an injury severity score of 16 or higher and a chest abbreviated injury scale (AIS) score of 3 or higher who were admitted to the emergency department of Hokkaido University Hospital from January 1, 2003, to December 31, 2016. Blush was defined as active extravasation of an intravascular contrast agent recognized on contrast-enhanced computed tomography. The date of trauma, trauma severity, treatments, and outcomes were obtained from the patients’ electronic medical records.

**Results:**

During the study period, 83 patients had severe lung contusions and 13 had blush. In-hospital mortality of patients with blush was significantly higher than that of patients without blush (53 vs. 10%, *P* < 0.001). Patients with blush required thoracic drainage more frequently (100 vs. 71%, *P* < 0.001) and support through mechanical ventilation more often (100 vs. 64%, *P* < 0.001) and for a longer duration (median duration, 0 vs. 25 days, *P* = 0.001) than patients without blush.

**Conclusions:**

Our study revealed that blush in lung contusions was not rare and was associated with a high risk of mortality in patients with severe blunt chest trauma. Clinicians should not hesitate to intervene if blush is detected in a lung contusion of a patient with blunt chest trauma.

## Introduction

For clinicians, reducing the number of preventable deaths among trauma patients in the emergency department is important. Chest trauma is the third most common cause of death in patients with multiple trauma, after abdominal and head trauma (1). Current studies report that the mortality rate of blunt chest trauma among patients with multiple trauma ranges from 5.5 to 25% (1–3). Severe lung contusion has a significant impact on clinical prognosis (2, 3). Severe lung contusions may also be associated with massive intrapulmonary bleeding, and severe cases requiring several clinical interventions are frequently reported (4, 5).

Blush, which has been frequently reported in hepatic and splenic trauma, is defined as active extravasation of the intravenous contrast agent recognized on computed tomography (6–8). Blush was reportedly observed in 6.6–17.1% of patients with severe hepatic and splenic trauma (6–11). The presence of blush in hepatic and splenic trauma indicates current progressive bleeding and implies the need for emergency hemostatic interventions such as transcatheter arterial embolization and abdominal surgery (6–11). Therefore, in some current management algorithms for hepatic and splenic trauma, blush has an important role in evaluating the necessity for emergency hemostatic interventions (6–11).

Although blush in lung contusions is sometimes observed in patients with severe blunt trauma in our clinical setting, its frequency and importance are unclear. Therefore, the present study was conducted to evaluate the frequency and relationship of blush in lung contusions with clinical outcomes in patients with blunt trauma.

## Materials and Methods

Approval for this study was obtained from the Institutional Review Board of the Ethics Committee at Hokkaido University Hospital. The ethics board waived the need for informed consent owing to the retrospective design.

### Patients

We retrospectively enrolled blunt injury patients with injury severity scores (ISS) ≥16 and chest abbreviated injury scale (AIS) scores ≥3 who were transported to the emergency department of Hokkaido University Hospital between January 1, 2003, and December 31, 2016. We excluded patients who had not undergone contrast-enhanced computed tomography (CECT) on admission to our emergency department. Furthermore, patients whose maximum diameter of lung contusion was less than 5 cm on computed tomography were excluded. Data on each patient’s age; sex; mechanism of injury; vital signs; respiratory Sequential Organ Failure Assessment, ISS, and AIS scores of each body part; in-hospital mortality; ventilator-free days; and specific treatments (such as mechanical ventilator support, thoracic drainage, isolated lung ventilation, extracorporeal membrane oxygenation support, and thoracic surgery) were obtained from the individual electronic medical records.

### Definition

**FIGURE 1:**
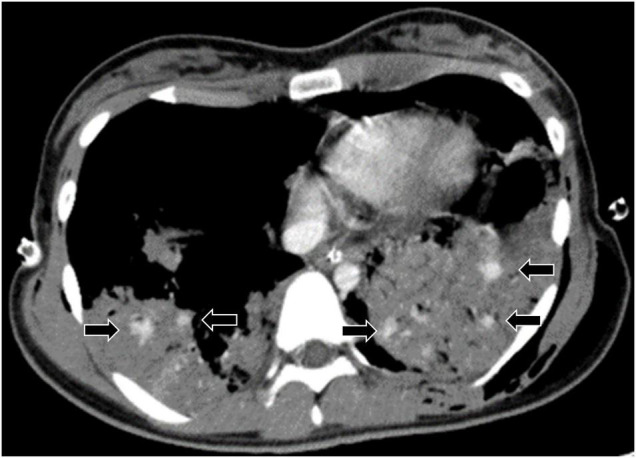
Typical blush in lung contusion. Arrows indicate extravasation of contrast media in the lung contusion, which is defined as blush.

Blush was defined as active extravasation of intravenous contrast recognized on CECT, similar to previous reports (6–8). A typical blush is shown in Figure 1. Severe lung contusion was defined as ≥5 cm of maximum diameter on computed tomography.

### Statistical Analysis

Statistical analysis was conducted using JMP 12.0.1 for Windows (SAS Institute Inc., Cary, NC, United States). Statistical significance was assessed at the 0.05 level unless otherwise noted. Data were summarized using medians and interquartile ranges (25th–75th percentile) or counts and percentages, where appropriate. Univariate comparisons were compared using chi-square tests for categorical variables and Wilcoxon rank-sum tests for continuous variables.

## Results

**FIGURE 2:**
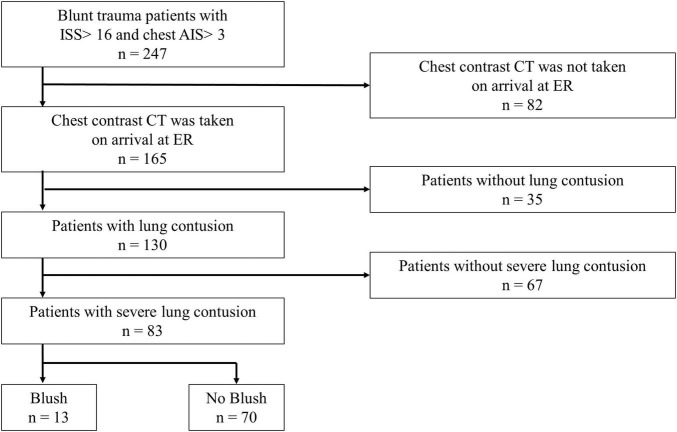
Flowchart of the study selection process. The blush group included patients in whom blush was detected in the severe lung contusion (≥5 cm of maximum diameter) on contrast-enhanced CT on arrival at the ER. The no blush group included patients who had severe lung contusions but for whom blush was not detected. ISS, injury severity score; AIS, abbreviated injury scale; CT, computed tomography; ER, emergency room.

From January 1, 2003, to December 31, 2016, 247 patients with ISS ≥16 and chest AIS scores ≥3 were admitted to the emergency department. Of these, 82 patients who had not undergone CECT on admission, 35 without lung contusion injuries, and 67 without severe lung contusion injuries were excluded. Among 83 patients with severe lung contusion injuries, 13 patients with blush in the lung contusions were identified (Figure 2). The patients with blush in the lung contusions were 10% of patients with lung contusions and 15% of patients with severe lung contusions.

The patients with blush did not show any difference in age, sex, or cause of injury from the patients without blush. There was no significant difference in individual chest AIS scores between the two groups; however, the abdomen AIS and ISS scores in patients with blush were higher than those in patients without blush (Table 1).

**TABLE 1:** Characteristics of patients.

	Blush *n* = 13	No blush *n* = 70	*P*-values
Age, years	36 (26–79)	38 (22–53)	0.302
Male, *n* (%)	10 (77)	50 (71)	0.690
**Cause of injury, *n* (%)**			
Fall	6 (46)	23 (33)	0.689
Traffic accident	7 (54)	40 (57)	
Other	0 (0)	7 (10)	
Glasgow coma scale	8 (3–11)	13 (6–15)	0.079
sBP, mmHg	111 (85–138)	117 (94–134)	0.408
Heart rate, bpm	113 (105–125)	96 (81–120)	0.481
Body temperature, °C	35.5 (35.4–36.3)	36.4 (35.7–36.9)	0.430
Pulmonary SOFA score in ER	2 (2–2)	2 (1–2)	0.048
Injury severity score	45 (29–48)	30 (25–38)	0.005
**Abbreviated injury score**			
Head/neck	4 (1–5)	3 (0–4)	0.175
Face	0 (0–2)	0 (0–0)	0.263
Chest	4 (4–4)	4 (4–4)	0.460
Abdomen	2 (2–4)	2 (0–2)	0.043
Extremity/pelvic	2 (1–3)	2 (0–3)	0.452
External	1 (0–1)	1 (0–1)	0.463

*sBP, systolic blood pressure; bpm, beats per minute; SOFA, sequential organ failure assessment; ER, emergency room.*

Tables 2, 3 show, respectively, the clinical interventions and outcomes of patients with and without blush. In patients with blush, the in-hospital and first 24-h mortality rates were significantly higher than those in patients without blush. Furthermore, the frequency of use of mechanical ventilator support in patients with blush was higher than that in patients without blush. Ventilator-free days in patients with blush were fewer than those for patients without blush. In terms of specific treatment, the use of isolated lung ventilation supports was frequent in patients with blush. In patients with blush, the major cause of death was severe intrathoracic bleeding, including respiratory failure induced by massive intrapulmonary bleeding (Table 4).

**TABLE 2:** Clinical interventions in patients with and without blush.

	Blush *n* = 13	No blush *n* = 70	*P*-values
Mechanical ventilator, *n* (%)	13 (100)	45 (64)	<0.001
Ventilator-free days, days	0 (0–19)	25 (15–28)	0.001
Isolated lung ventilation, *n* (%)	3 (23)	2 (2.9)	0.125
Thoracic drainage, *n* (%)	13 (100)	50 (71)	<0.001
ECMO support, *n* (%)	1 (7.8)	2 (2.9)	0.553
Thoracic surgery, *n* (%)	3 (23)	10 (14)	0.505

*ECMO, extracorporeal membrane oxygenation.*

**TABLE 3:** Clinical outcomes in patients with and without blush.

	Blush *n* = 13	No blush *n* = 70	*P*-values
Mortality rate			
First 24 h, *n* (%)	7 (53)	4 (6)	<0.001
In-hospital, *n* (%)	7 (53)	7 (10)	<0.001
Length of hospital stay, days	4 (1–27)	27 (15–45)	0.027

**TABLE 4:** Cause of death in patients with and without blush.

	Blush *n* = 7	No blush *n* = 7
Bleeding		
Intrathoracic bleeding*	5	0
Intraabdominal bleeding	1	2
Retroperitoneal bleeding	1	0
Brain trauma	0	3
Other	0	2

*Including respiratory failure induced by massive intrapulmonary bleeding; P = 0.016 by the chi-square test.

## Discussion

In this single-institute retrospective observational study, blush in lung contusions occurred in 15% of patients with severe lung contusions. Furthermore, the blush in lung contusions was associated with the necessity for various interventions and with a high mortality rate. In addition, the blush group had more acute deaths than the no blush group. Therefore, the length of hospital stay in the blush group was shorter than that in the no blush group because of survival bias.

Treatment strategies have been established for blush in hepatic and splenic trauma, and the mortality rate of these patients has been reported to be 0–8.4% (6, 8–10). However, to the best of our knowledge, no investigation into the urgency of blush in lung contusion has previously been reported. In the present study, blush in the lung contusion was more frequently observed than in previous hepatic and splenic trauma (6, 8–10). Furthermore, blush in lung contusions was clearly associated with poor patient outcomes. However, the urgency of seeing blush in a lung contusion CECT had not been recognized by attending physicians in our institution before the present study. Therefore, because no management strategy had also been established, patients with blush in lung contusion would have a high mortality rate. Similar to blush in hepatic or splenic trauma, blush in lung contusion will be a good indicator to guide the management strategy for patients with chest trauma.

Blush in lung contusions indicates ongoing hemorrhage, similar to blush in hepatic and splenic injuries. However, the effects of ongoing hemorrhages are much different between lung contusions and hepatic/splenic injuries, because the hemorrhages in lung contusions are also frequently intra-airway hemorrhages. Intra-airway hemorrhages from lung contusions exacerbate respiratory conditions, even if the amount of hemorrhage is small (4, 12, 13). Therefore, when blush is detected in lung contusions, clinicians need to intervene more aggressively to stop the hemorrhage.

Therapeutic interventions for hemorrhage in lung contusions are twofold: protection of the contralateral lung from pulmonary hemorrhage and hemostatic intervention (13). One simple form of protection of the contralateral lung from intra-airway hemorrhage is isolated lung ventilation. When blush in the lung contusion is detected, it may be necessary to isolate the contralateral lung before the exacerbation of the respiratory condition by intra-airway hemorrhage from the lung injury. Although lung resection and lobectomy are definitive hemostatic procedures for lung injury, they are highly invasive. Therefore, *trans*-arterial embolization is frequently selected for pulmonary hemorrhage, similar to the treatment of hepatic and splenic injuries (13, 14). However, the lungs have blood supply from two vessel systems: the tracheal and pulmonary artery systems. Furthermore, blood flow in the pulmonary artery is more than that in the tracheal artery in the lung. Therefore, control of blood flow in the pulmonary artery by, for example, balloon occlusion, is necessary to stop the pulmonary hemorrhage (15, 16). When blush in lung contusions is detected, there is potential to improve prognosis by actively protecting the contralateral lung and performing hemostatic interventions to prevent further bleeding and worsening of the respiratory status.

### Strengths and Limitations

The strength of our study lies in the fact that it is the first one to report the frequency of blush in lung contusions and its relationship with the outcomes. However, there are inherent limitations in any retrospective evaluation. Additionally, the numbers in this study may be considered small, hence precluding broad generalization. Furthermore, the observation period was so long that the treatment algorithm might have changed and contributed to the patients’ outcomes. Also, the significant difference in in-hospital stay between groups was affected by survival bias. However, to the best of our knowledge, this is the first report about blush in lung contusions and its characteristics. More extensive research is needed to confirm our results.

Our study revealed that blush in lung contusions was not rare and was associated with the necessity for various interventions and a high mortality rate. When blush in lung contusions is detected in patients with blunt chest trauma, clinicians must be aware of the dangers of the sign. Immediate protection of the contralateral lung from pulmonary hemorrhage and performing hemostatic interventions may improve outcomes, but further large-scale.

## Data Availability Statement

The original contributions presented in the study are included in the article/supplementary material, further inquiries can be directed to the corresponding author.

## Ethics Statement

This study was approved by Hokkaido University Hospital, and the requirement for informed consent was waived.

## Author Contributions

NT collected and interpreted the data and drafted the manuscript. MH conceived the study, analyzed and interpreted the data, and drafted the manuscript. SY read the manuscript and revised it for important intellectual content. All authors read and approved the final manuscript.

## Conflict of Interest

The authors declare that the research was conducted in the absence of any commercial or financial relationships that could be construed as a potential conflict of interest.

## Publisher’s Note

All claims expressed in this article are solely those of the authors and do not necessarily represent those of their affiliated organizations, or those of the publisher, the editors and the reviewers. Any product that may be evaluated in this article, or claim that may be made by its manufacturer, is not guaranteed or endorsed by the publisher.
